# Multiple Myeloma Includes Phenotypically Defined Subsets of Clonotypic CD20+ B Cells that Persist During Treatment with Rituximab

**DOI:** 10.4137/cmo.s615

**Published:** 2008-03-27

**Authors:** Linda M. Pilarski, Eva Baigorri, Michael J. Mant, Patrick M. Pilarski, Penelope Adamson, Heddy Zola, Andrew R. Belch

**Affiliations:** 1 Department of Oncology, University of Alberta and Cross Cancer Institute, 11560 University Avenue, Edmonton AB T6G1Z2, Canada; 2 Department of Medicine, University of Alberta, Edmonton AB, T6G2R7, Canada; 3 Department of Electrical and Computer Engineering, University of Alberta, Edmonton AB T6G2V4, Canada; 4 Child Health Research Institute, 72 King William Road, North Adelaide SA 5006, Australia

**Keywords:** multiple myeloma, B lineage cells, CD19 epitopes, CD20 epitopes, B cell depletion by rituximab

## Abstract

Potential progenitor B cell compartments in multiple myeloma (MM) are clinically important. MM B cells and some circulating MM plasma cells express CD20, predicting their clearance by treatment with anti-CD20. Here we describe two types of clonotypic CD20+ B cell in peripheral blood of myeloma patients, identified by their expression of CD19 and CD20 epitopes, their expression of CD45RA and their light scatter properties. Thus, the circulating component of the MM clone includes at least two distinct CD19+ CD20+ B cell compartments, as well as CD138+ CD20+ plasma cells. To determine whether either or both B cell subsets and the CD20+ plasma cell subset were depleted by anti-CD20 therapy, they were evaluated before, during and after treatment of patients with rituximab (anti-CD20), followed by quantifying B cell subsets over a 5 month period during and after treatment. Overall, all three types of circulating B lineage cells persist despite treatment with rituximab. The inability of rituximab to prolong survival in MM may result from this failure to deplete CD20+ B and plasma cells in MM.

## Introduction

The malignant clone in multiple myeloma is complex and highly heterogeneous in the bone marrow and the blood. Although myeloma plasma cells cause most pathological symptoms and are the only compartment of the malignancy that is routinely monitored during treatment, earlier B lineage compartments persist throughout therapy and are likely sources of relapse. In multiple myeloma, the clonotypic IgH VDJ gene rearrangement provides a unique molecular signature that remains constant throughout the course of disease. Other genetic and/or molecular signatures characterize MM, but these are usually expressed by only a subset of the MM plasma cells, indicating heterogeneity within the MM clone and compromising the use of these signatures as markers to monitor disease burden or to detect presumptive progenitor compartments of the disease that escape therapy. The IgH VDJ provides an unequivocal marker for all cellular compartments of MM, that is unaffected by any known selective pressures and exists independently of differentiation stage, cell morphology, phenotypic characteristics or acquisition of more aggressive genotypes.

A variety of work has shown that MM includes clonotypic B lineage cells at stages earlier than the compartment of malignant plasma cells in the bone marrow (Bakkus et al. 1994; Bergsagel et al. 1995; Pilarski et al. 1996; Szczepek et al. 1997; Szczepek et al. 1998; Pilarski et al. 2000a). Our previous work has definitively shown that the peripheral blood of MM patients is colonized by CD19+ clonotypic B cells (Szczepek, Seeberger, Wizniak, Mant, Belch, and Pilarski, 1998), as well as by clonotypic IgM+ B cells (Reiman et al. 2001; Taylor et al. 2002). The majority of cells in this B lineage compartment of clonotypic B cells have a monocytoid light scatter pattern characteristic of large granular cells, phenotypic characteristics of activated lymphocytes (Bergsagel, Masellis, Szczepek, Mant, Belch, and Pilarski, 1995), constitutive cytokine synthesis (Szczepek, Belch, and Pilarski, 2001) and migratory properties consistent with a compartment capable of mediating malignant spread (Masellis-Smith et al. 1996). The activated clonotypic B cell compartment is frequent in peripheral blood (Szczepek, Seeberger, Wizniak, Mant, Belch, and Pilarski, 1998). The characteristics of less frequent resting B cell compartments and the frequency of clonotypic cells within these compartments remains as yet uncharacterized.

Xenografted peripheral blood cells harboring circulating malignant B cells but lacking detectable plasma cells are able to xenograft human MM to immunodeficient mice, as can leukemic plasma cells and CD34+ progenitor fractions of MM mobilized blood autografts (Pilarski et al. 2002; Pilarski et al. 2000b; Pilarski and Belch, 2002; Reiman, Seeberger, Taylor, Szczepek, Hansen, Mant, Coupland, Belch, and Pilarski, 2001). Overall, precursor function within the MM clone appears to be complex, involving multiple components of the MM hierarchy, including both B and plasma cells (Pilarski et al. 2004). This concept is supported by recent work from Matsui et al. (Matsui et al. 2004) showed that CD34-CD20 + MM cell fractions of colony forming cells gave rise to engraftment in vivo, who and were sensitive to rituximab in vitro. However, the significance of this work is uncertain because no confirmation of the MM clonotypic signature was carried out. In non-leukemic stages of disease, infrequent plasma cells can be detected in peripheral blood of MM patients, having an apparently abnormal phenotype and monotypic light chain expression (Witzig et al. 1993; Witzig et al. 1996), but their generative capabilities and their clonotypic relationship to MM remain unexplored.

The objective of the current study was to identify B cell compartments of the MM clone, determine the frequency of clonotypic B cells among their normal counterparts, and evaluate the impact of rituximab therapy (anti-CD20 serotherapy) on circulating clonotypic B and plasma cells in myeloma. Unlike B cells in non-Hodgkin’s lymphoma and B-CLL, all compartments of CD20+ B lineage cells, including a set of circulating CD20+ plasma cells, persist throughout rituximab therapy despite their medium to high density expression of CD20. This suggests that in vivo, antibody targeting by rituximab is ineffective against CD20+ clonotypic or presumptively clonotypic compartments of the MM clone.

## Methods

### Patients and samples

After approval from the University of Alberta Health Research Ethics Board and informed consent, a total of 118 patients with multiple myeloma donated heparinized blood samples for analysis. Six patients were treated with rituximab as previously described (Treon et al. 2002), with blood samples for analysis taken pre-treatment, prior to each of 4 weekly infusions of rituximab and at sequential time points up to 5 months after initiating the treatment. Peripheral blood mono-nuclear cells were prepared as previously described (Szczepek, Seeberger, Wizniak, Mant, Belch, and Pilarski, 1998) and phenotypically analyzed as described in methods.

### Reagents

Pan CD19 antibodies were B4-FITC (Coulter Corp. (Miami FL) and custom conjugated FMC63-FITC prepared from ascites fluid as previously described (Szczepek, Seeberger, Wizniak, Mant, Belch, and Pilarski, 1998). Leu12-FITC (CD19) and CD3-PE were from Becton Dickinson (San Jose CA), CD138-PE from Immunotec (Marseille France), and CD20-PE (B1) from Coulter Corp. Rituximab-FITC was custom conjugated using aliquots of rituximab from the Cross Cancer Institute. CD45RA-FITC (FMC44) was custom conjugated (Pilarski and Deans, 1993). Isotype controls from Becton Dickinson were FITC or PE labeled as required. Staining of PBMC with conjugated antibodies and analysis by flow cytometry was as previously described (Szczepek, Seeberger, Wizniak, Mant, Belch, and Pilarski, 1998). Neuraminidase was from Sigma Aldrich Canada Ltd. (Oakville ON). PBMC were treated at 37 ^o^C for 1 hour with 0.5 u/ml (100 μl/well), followed by washing and staining with the indicated antibodies. For molecular analysis, MM PBMC were stained with Leu12-FITC and sorted as previously described (Szczepek, Bergsagel, Axelsson, Brown, Belch, and Pilarski, 1997; Szczepek, Seeberger, Wizniak, Mant, Belch, and Pilarski, 1998) to provide purified populations of Leu12+ B cells.

A CD19 transfectant was prepared by cloning a full-length CD19 cDNA into *E. coli* XL1- blue and transfecting it in CHO-K1 cells (PJ Adamson, PhD thesis, University of Adelaide, 2000). Transfectants were stained with CD19 antibodies to confirm their specificity for CD19.

### In situ RT-PCR

Clonotypic IgH VDJ sequences for the MM clone from each of 15 MM patients were derived and validated by confirming that the selected sequence was expressed by the majority of autologous BM plasma cells, followed by design and testing of patient specific primers annealing to CDR2 and CDR3 as previously described (Szczepek, Seeberger, Wizniak, Mant, Belch, and Pilarski, 1998). Sorted FMC63+Leu12+or FMC63+Leu12−PBMC were placed on slides, prepared for in situ RT-PCR and then in situ RT-PCR was performed as described previously (Pilarski, Giannakopoulos, Szczepek, Masellis, Mant, and Belch, 2000a; Szczepek, Seeberger, Wizniak, Mant, Belch, and Pilarski, 1998; Szczepek, Bergsagel, Axelsson, Brown, Belch, and Pilarski, 1997). All experiments included specificity controls and controls for RNA integrity as detailed in previous papers (Szczepek, Bergsagel, Axelsson, Brown, Belch, and Pilarski, 1997; Szczepek, Seeberger, Wizniak, Mant, Belch, and Pilarski, 1998). The number of sorted PBMC expressing the clonotypic transcripts were compared on a parallel slide to the number with transcripts for histone, to provide a % value.

### Rituximab therapy

Patients were in a previously described cohort (Treon, Pilarski, Belch, Shima, Szczepek, Raje, Hideshima, Chauhan, Tau, Davies, Preffer, and Anderson, 2002). Blood samples were obtained at day 0 and day 4 for each cycle of therapy, and at monthly intervals after completion of 4 cycles of rituximab (10–12 blood samples per patient). CD20+ B cells that may have bound rituximab remain detectable by their expression of CD19.

## Results

### Circulating PBMC from MM patients include two distinct subsets of CD19+ B cells

**Figure 1:**
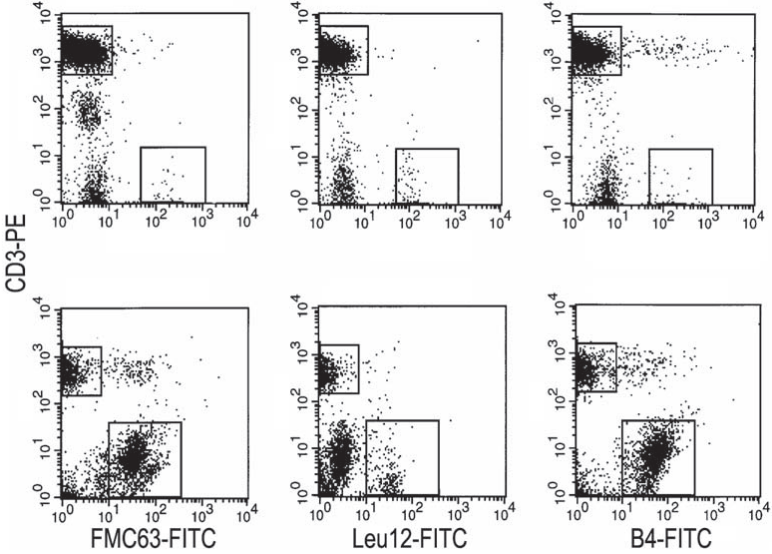
Expression of CD19 Epitopes on B cell populations in multiple myeloma and in healthy donors: Absence of the Leu12 epitope on most MM B cells PBMC from a representative healthy donor (top row of panels) and a representative MM patient (bottom row) were stained in two color immunofluoresence with CD3-PE and three types of CD19-FITC, B4, FMC63 and Leu12 as indicated in methods. The mean values for each antibody are given in Table 1. Files were ungated. Sorted B cells stained as shown here, and sorted within the analysis gates shown in this figure have been shown to express intracellular immunoglobulin, CD19 transcripts and IgH transcripts (Pilarski, Giannakopoulos, Szczepek, Masellis, Mant, and Belch, 2000a; Szczepek, Bergsagel, Axelsson, Brown, Belch, and Pilarski, 1997) as well as clonotypic IgH VDJ (Pilarski, Giannakopoulos, Szczepek, Masellis, Mant, and Belch, 2000a; Szczepek, Bergsagel, Axelsson, Brown, Belch, and Pilarski, 1997; Szczepek, Seeberger, Wizniak, Mant, Belch, and Pilarski, 1998).

**Figure 2:**
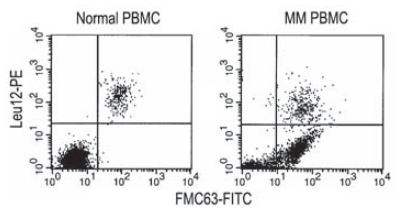
Two distinct populations of B cells in MM patients Respresentative staining of PBMC from a healthy donor and an MM patient in two color immunofluorescence with Leu12-PE and FMC63-FITC, showing two distinct populations of B cells in MM patients (CD19+ Leu12− and CD19+ Leu12+) but only one in healthy donors (CD19+Leu12+). Previous work has shown that the Leu12− populations detected by B4 and FMC63 are B cells and express clonotypic transcripts (Pilarski, Giannakopoulos, Szczepek, Masellis, Mant, and Belch 2000a; Pilarski, Szczepek, and Belch 1997; Szczepek, Seeberger, Wizniak, Mant, Belch, and Pilarski 1998; Szczepek, Bergsagel, Axelsson, Brown, Belch, and Pilarski 1997).

Previous work has reported the presence of an abnormally high percent of B cells (10%–30%) in PBMC from patients with MM when detected with anti-CD19 mAbs B4 or FMC63 (Bergsagel, Masellis, Szczepek, Mant, Belch, and Pilarski, 1995; Szczepek, Seeberger, Wizniak, Mant, Belch, and Pilarski, 1998) but a low % when detected with the Leu12 mAb (Kay et al. 1997). To resolve the apparent discrepancy, MM PBMC were analyzed for CD19 expression using all three mAbs (Fig. 1 and Table 1). Figure 1 shows representative PBMC from a healthy donor (top row) and from an MM patient (bottom row). As previously reported (Bergsagel, Masellis, Szczepek, Mant, Belch, and Pilarski, 1995; Pilarski, Pruski, and Belch, 2002), after staining for the epitopes recognized by anti-CD19 mAbs B4 and FMC63, a large population of B cells was detectable in myeloma patients but not in healthy donors. Although it has been suggested that for MM PBMC much of the staining with B4 and FMC63 is a serum-related artifact (Rasmussen, Jensen, and Johnsen, 2000), no evidence was provided to indicate whether the involved cells did or did not express CD19 transcripts, nor whether they expressed IgH transcripts and synthesized IgH. In contrast, our previous work has clearly shown these cells to express CD19, IgH and clonotypic VDJ transcripts (Pilarski, Giannakopoulos, Szczepek, Masellis, Mant, and Belch, 2000a; Szczepek, Bergsagel, Axelsson, Brown, Belch, and Pilarski, 1997; Szczepek, Seeberger, Wizniak, Mant, Belch, and Pilarski, 1998) as well as synthesizing IgH immunoglobulin (Szczepek, Bergsagel, Axelsson, Brown, Belch, and Pilarski, 1997), validating their identification as bona fide CD19+ expressing B cells, and confirming their clonal relationship to autologous MM plasma cells. However staining of the same PBMC samples with the Leu 12 anti-CD19 mAb detected only a small population of B cells in either healthy donors or MM patients. Overall, for healthy donors, each of the three mAbs detected the same % of B cells in any given PBMC sample (6%–7%). This was not true for MM patients. Although a comparable % of B cells were detected with FMC63 and B4, respectively a mean of 27% or 30% of PMBC, Leu12 detected only 2%–7% B cells in MM PBMC. Like FMC63/B4+ MM B cells, Leu12+ MM B cells escape chemotherapy, remaining detectable during and after treatment (Table 1). Two color staining with Leu12 and FMC63 revealed two distinct subsets of B cells in MM PBMC, those expressing both epitopes, and those expressing only the FMC63 epitope (Fig. 2); only the former subset is detected in PBMC of healthy donors.

### All three CD19 mAbs detect CD19 on CD19 transfectants

**Figure 3:**
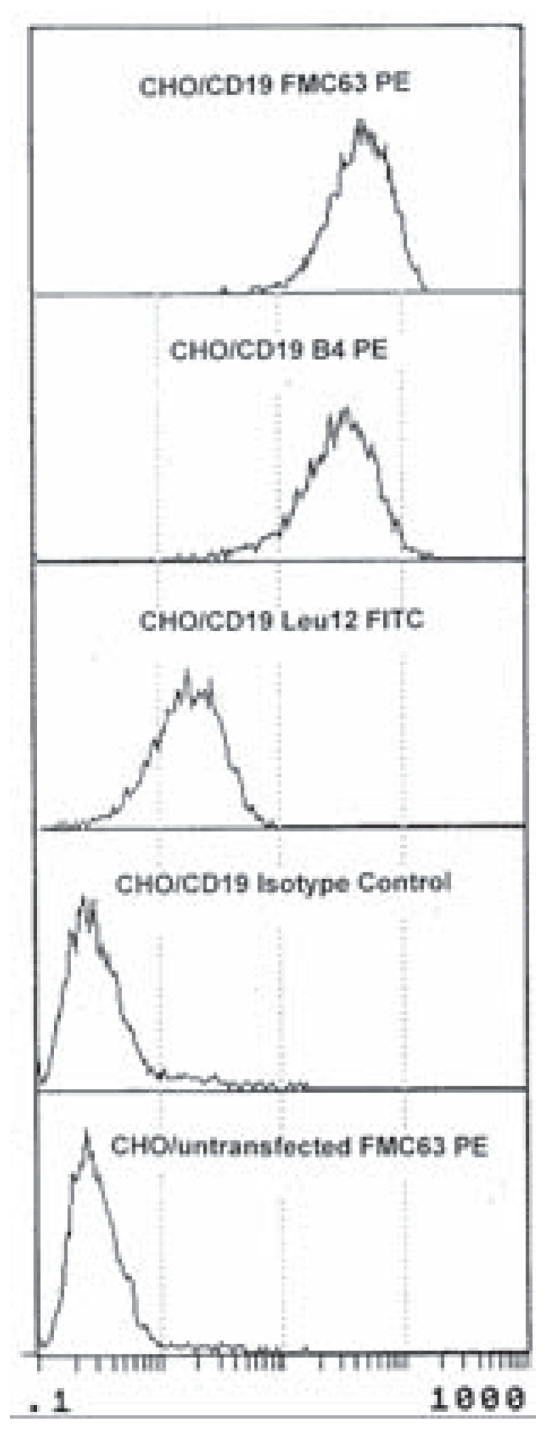
Expression of CD19 epitopes on CD19 transfectants To confirm that all three antibodies detected CD19 epitopes, FMC63, B4 and Leu12 antibodies were used to stain CD19 transfectants. The transfectants were shown to express the CD19 epitope as detected by binding of each antibody. Appropriate isotype controls were performed for each anti-CD19 antibody. The observed staining with Leu12 indicates that in transfectants, unlike MM cells but similar to B cells from healthy donors, the Leu12 epitope is exposed.

To confirm that all three preparations of anti-CD19 mAbs were specific for CD19, stable CD19 CHO cell transfectants were stained and analyzed (Fig. 3). All three mAbs detected CD19 on the transfected cells. This analysis further confirms previous work showing that sorted CD19 MM B cells stained with FMC63 express CD19 transcripts (Pilarski, Giannakopoulos, Szczepek, Masellis, Mant, and Belch, 2000a; Szczepek, Bergsagel, Axelsson, Brown, Belch, and Pilarski, 1997).

### On MM B cells, an otherwise cryptic Leu12 epitope is revealed by neuraminidase treatment

**Figure 4:**
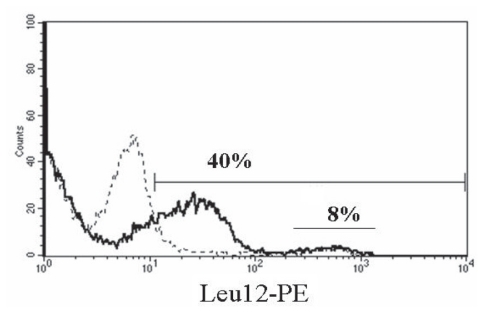
Neuraminidase treatment reveals Leu12 epitopes on MM B cells MM PBMC were treated with neuraminidase (solid line) or left untreated (dashed line) as described in methods, followed by staining with Leu12-PE. The marker bar indicates staining above negative control values. The numerical value above the marker bar reports the number of Leu12+ cells after neuraminidase treatment. The Leu12^bright^ peak between 10^2^ and 10^3^ represents expression that is independent of neuraminidase treatment (dotted line), while the lower intensity peak represents Leu12 epitopes revealed by neuraminidase (solid line). PBMC were also stained before and after treatment with fluorescent conjugates of FMC63, B4, CD20 mAbs B1 and rituximab; the staining profiles were comparable before and after treatment for all antibodies except Leu12.

The Leu12 epitope could be masked by other cell surface proteins on MM B cells but not on B cells from healthy donors. MM B cells, but not B cells from healthy donors, have been shown to express a high density of Muc1 (Treon et al. 1999), a branched sialoprotein that may obscure some cell surface epitopes. To determine whether the apparent absence of the Leu12 epitope resulted from masking by the Muc1 or other sialoproteins, MM PBMC from three patients were treated with neuraminidase, an enzyme that cleaves the terminal sugar group and has been shown to reveal cryptic epitopes (Tetteroo et al. 1984). Neuraminidase treatment reveals an otherwise cryptic Leu12 epitope. A representative patient sample is shown in Figure 4. Prior to neuraminidase treatment, 8% of the MM PBMC were Leu12+. After treatment, 40% of the PBMC were Leu12+; these cells co-expressed FMC63, B4, B1 and rituximab epitopes, and the majority had high light scattering properties as described below. The percent of MM PBMC expressing FMC63/B4 (CD19) or B1/rituximab (CD20) epitopes did not change after neuraminidase treatment. The same pattern was observed for all three treated MM PBMC samples. Thus, the use of FMC63, B4 or CD20 antibodies allows detection of the aggregate population of Bcells in MMPBMC, including both the Leu12+ cells and the Leu12– B cells, without the need to introduce neuraminidase. This work confirms that both subsets of MM B cells are detectable with all three anti-CD19 antibodies, but that in the absence of enzyme treatment to expose them, Leu12 epitopes are cryptic on the large MM B cells. The expression by both subsets of rituximab epitopes suggests that they should be susceptible to treatment with rituximab.

### Leu12+ MM B cells are small resting lymphocytes that coexpress CD20 and CD45RA

**Figure 5:**
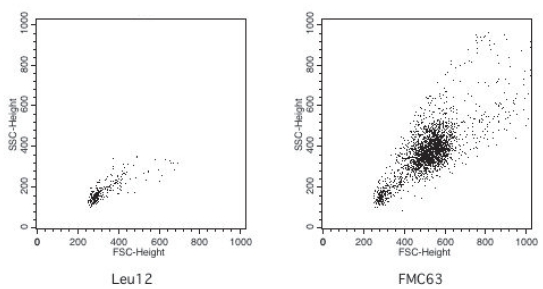
Leu12+ B cells have low forward and side scatter, in contrast to FMC63+ B cells which include a subpopulation with high forward and side scatter MM PBMC were stained with Leu12 or FMC63. Files were gated on the CD19+ population for each antibody: Leu12+ = 3.3% of PBMC and FMC63+ = 29% OF PBMC. B4+ B cells had the same scatter profile as did FMC63+ B cells. B1+ or rituximab+ B cells also have a scatter profile comparable to that of the FMC63+ B cells. The vertical axis represents side scatter (SSC) and the horizontal axis represents forward scatter (FSC). The horizontal axes from left to right represent increasing extent of forward scatter. The left panel shows a single population of Leu12+ B cells having low side and forward scatter. The right panel shows the two subpopulations distinguished by FMC63 (FMC63+ high side or forward scatter, and FMC63+ low side or forward scatter).

**Figure 6:**
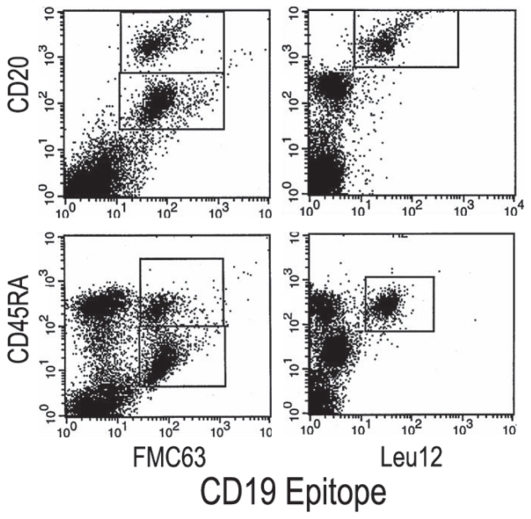
Phenotypic analysis of CD19, CD20 and CD45RA identify two distinct populations of CD19+20+ B cells Representative MM PBMC were stained with anti-CD19-FITC (horizontal axis) and either CD20-PE vertical axis, top row) or CD45RA-PE (vertical axis, bottom row). The boxes indicate subopulations defined by this analysis. The left top panel shows FMC63+ CD20^Moderate^, and FMC63+CD20^bright^ subpopulations, and the left bottom panel shows FMC63+CD45RA^bright^ and FMC63+CD45RA^dim^ populations. The right panels show a single population identified by Leu12.

Flow cytometry analysis indicated that Leu12+ MM B cells have low forward and side scatter properties (Fig. 5, left) that are comparable to the known light scatter characteristics of healthy donor B cells. On average, 83+/−4% of Leu12+ MM B cells are small lymphocytes. In contrast, the FMC63+ MM B cells include a majority compartment that has high forward and side scatter (Fig. 5, right). The Leu12+ and FMC63+ (Leu 12−) MM B cells also differ in their expression of CD20 and CD45RA. Multicolor flow cytometry shows that FMC63+ B cells include two compartments of B cells, a set with a high density of CD20 and high expression of CD45RA, and a more frequent set having moderate CD20 expression and dim CD45RA (Fig. 6, left panels). The Leu12+ MM B cells have high CD20 and high CD45RA (Fig. 6, right panels), comparable to that of healthy donor B cells ((Jensen et al. 1989) and not shown). The staining profile of the Leu12+ B cells indicates that they are the same cells that appear as the minority compartment of the FMC63+ MM B cells. The distinct phenotypic characteristics of the two B cell subsets means that combined analysis of CD19 and/or CD20 with light scatter characteristics provides useful clinical phenotype to monitor their presence in peripheral blood of MM patients.

### A proportion of the Leu12+ MM B cells are clonotypic

**Figure 7:**
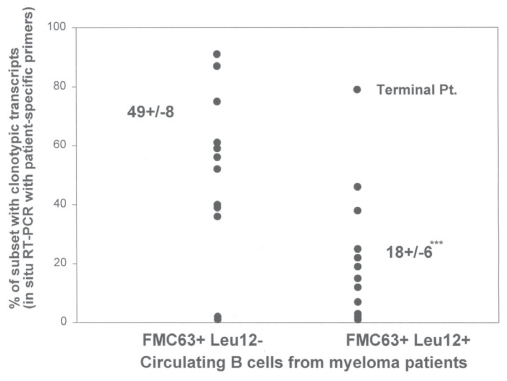
A proportion of Leu12+ MM B cells are clonotypic FMC63+Leu12− (monocytoid gates) and FMC63+Leu12+ (lymphocyte gates) B cells were sorted and analyzed for clonotypic IgH VDJ transcripts using in situ RT-PCR. Leu12− monocytoid B cells were sorted from PBMC of 12 MM patients. Leu12+ lymphocyic B cells were sorted from PBMC of 15 MM patients. Leu12 negative B cells included significantly more clonotypic B cells than did Leu12+ B cells (p = .003). One of the patients analyzed for clonotypic Leu12+ B cells was in a terminal stage of disease.

Previous work has shown that the majority of the FMC63+ MM B cells express clonotypic IgH VDJ and CD19 transcripts (Pilarski, Giannakopoulos, Szczepek, Masellis, Mant, and Belch, 2000a; Pilarski, Szczepek, and Belch, 1997; Szczepek, Seeberger, Wizniak, Mant, Belch, and Pilarski, 1998; Szczepek, Bergsagel, Axelsson, Brown, Belch, and Pilarski, 1997); most of these are Leu12-. To determine their involvement in the MM clone, Leu12+ MM B cells were sorted from PBMC of 15 MM patients who were on intermittant chemotherapy or had discontinued therapy. In situ RT-PCR was used to determine the number having transcripts that were amplified using patient-specific primers annealing to CDR2 and CDR3 of the clonotypic IgH VDJ sequence. On average, 18+/−6% of Leu12+ MM B cells are clonotypic (range 0%–79%) (Fig. 7). Intriguingly, the seven MM patients having fewer than 1% Leu12+ B cells in PBMC had the highest proportion that were clonotypic (36+/−13%), while the eight MM patients with >1% Leu12 + MM B cells in PBMC had a reduced proportion that were clonotypic (9+/−3%) (p = 0.01).

### Rituximab therapy fails to deplete circulating CD20+ MM B cell subsets or plasma cells

Previous work has shown that rituximab appears to target CD20+ plasma cells in the BM, but otherwise has little clinical impact (Treon, Pilarski, Belch, Shima, Szczepek, Raje, Hideshima, Chauhan, Tau, Davies, Preffer, and Anderson, 2002). This and other work shows that both identified subsets of clonotypic MM B cells express CD19 as well as expressing CD20 at moderate or high intensity, suggesting that they should be targeted by rituximab. To determine whether or not this was occurring, we analyzed longitudinal PBMC samples from 6 patients who had been treated with rituximab. B cells were detected by staining with both anti-CD20 and anti-CD19 antibodies. For this analysis, CD19/CD20 expression and light scatter properties were used as surrogate markers to identify the two subsets of clonotypic MM B cells described above. PBMC were stained in two color immunofluorescence with both anti-CD20 and anti-CD19, to ensure detection of any B cells whose CD20 epitopes were masked by circulating rituximab. Prior to treatment, all CD20+ B cells co-expressed CD19. Ten to twelve sequential blood samples were analyzed for each of the 6 patients treated with rituximab. Pre-treatment, all B cells in MM PBMC co-expressed both CD19 and CD20, as do B cells from healthy donors. B cells in MM PBMC were evaluated at sequential time points during and after rituximab treatment of 6 MM patients. Both large (high forward and side scatter properties: monoctytoid gates) and small (low forward and side scatter: lymphocyte gates) subsets of CD19+ CD20+ B cells in MM PBMC, as identified by either anti-CD19 (Table 2) or anti-CD20 (Table 3), resist serotherapy with anti-CD20. It is interesting to note that there was only transient depletion of the predominantly normal CD20^hi^ subset that on average is only 18% clonotypic. Although patients received 4 cycles of rituximab, for most patients the % of circulating B cells did not consistently decrease over the 5 month study period. Analysis was performed before and after each treatment with rituximab and then at monthly intervals. For both compartments of B cells described above, the % of small and large CD20+ cells before, over the course of treatment and after therapy is detailed in Table 3. Absolute numbers are given in Table 4. Based on the specific effects of rituximab on B cells, as measured by the % of CD20+ cells in PBMC, for all patients a transient decrease was detected in one or more samples. However, for 5/6 patients, the % of CD20+ B cells in both compartments were also transiently or persistently increased during and after the therapy. 2/6 patients had substantial post-rituximab increases in the compartment of small CD20^bright^ B cells (a subset comparable to that shown in Figs. 5 and 6). Furthermore, circulating CD138+CD20+ plasma cells were also resistant to four cycles of anti-CD20 serotherapy (Table 2). When the absolute number of B cells was evaluated, taking into consideration non-specific changes in the number of lymphocytes, there was a minor increase or slight decrease in 4/6 patients and a decrease of about 2–4 fold in 2/6 patients. However circulating B lineage cells remained readily detectable in all patients, indicating the failure of rituximab to clear the majority of either the large CD20^med^ or small CD20^bright^ MM B cells described above, or the CD20+ circulating plasma cells.

## Discussion

This work demonstrates the existence of two distinct B cell compartments in the peripheral blood of MM patients that include cells expressing the MM clonotypic signature. Both B cell compartments escape conventional chemotherapy, implicating them in the almost universal relapse that afflicts MM patients. This work shows that they also escape treatment with rituximab despite their expression of CD20. Previous work has confirmed the existence of a numerically abnormal B cell compartment with an activated B cell phenotype and, as shown here, no detectable Leu12 CD19 epitope. These activated MM B cells express the MM clonotypic IgH VDJ clonotypic signature, IgH and CD19 transcripts (Pilarski, Giannakopoulos, Szczepek, Masellis, Mant, and Belch, 2000a; Szczepek, Bergsagel, Axelsson, Brown, Belch, and Pilarski, 1997; Szczepek, Seeberger, Wizniak, Mant, Belch, and Pilarski, 1998), validating them as bona fide B cells with intrinsic expression of CD19 and clonotypic IgH. MM B cells xenograft MM to immunodeficient mice (Pilarski, Seeberger, Keats, Taylor, Coupland, and Belch, 2002; Pilarski, Hipperson, Seeberger, Pruski, Coupland, and Belch, 2000b; Pilarski and Belch, 2002). The present study shows the presence of an MM B cell compartment with a phenotypic profile comparable to that of healthy donors. However, this phenotypically normal B cell compartment includes a mean of 18% clonotypic cells, with occasionally as many as 79% clonotypic cells, confirming a relationship with the malignant clone. More clonotypic cells are detected among the Leu12+ population in patients with fewer than 1% Leu12+ B cells, than are found among those patients having >1% Leu12 + B cells. As the aggregate number of B cells is reduced by chemotherapy, polyclonal populations of presumptively normal B cells are preferentially depleted while the malignant MM B cells are preferentially enriched. Our previous work also demonstrated significant reductions in polyclonal B cells and their functional capabilities (Pilarski et al. 1984; Pilarski et al. 1986; Pilarski, Ruether, and Mant, 1985). This may explain the observations of Kay et al. who found that infectious episodes increased in MM patients having low numbers of Leu12+ B cells (Kay, Leong, Kyle, Greipp, Billadeau, Van Ness, Bone, and Oken, 1997), likely reflecting this preferential loss of polyclonal B cells. Since polyclonal B cells are required to fight infections, their relative absence may also partially explain the humoral immune deficiency in MM (Cone and Uhr, 1964) and the reduced levels of polyclonal immunoglobulins.

The MM compartment of “activated” B cells lacks the Leu12 epitope of CD19. Operationally, this means that use of the Leu12 antibody detects only the “resting” compartment of circulating B cells in MM patients, and excludes detection of the majority compartment of clonotypic “activated” B cells with monocytoid scatter properties. Neuraminidase digestion to remove terminal sialic acid residues revealed Leu12 epitopes on these activated B cells. This may reflect expression of sialic acid residues on CD19 itself or on neighboring proteins such as Muc1, that would otherwise mask the Leu12 epitope. Alternatively, neuraminidase treatment may alter the surface charge of the cell leading to increased exposure of the Leu12 epitope to its antibody. Previous work indicated that MM B cells having a low scatter profile, shown here to be Leu12+, had reduced levels of the Muc1 sialoprotein as compared to the activated set of MM B cells (not shown). Furthermore, the activated clonotypic B cells in MM express the CD45R0 isoform (Jensen et al. 1991) which in healthy donors identifies late stage B cells (Jensen, Poppema, Mant, and Pilarski, 1989). In contrast, the Leu12 + MM B cells express the CD45RA isoform which is also expressed on essentially all circulating B cells in healthy donors (Jensen, Poppema, Mant, and Pilarski, 1989), further confirming that they are resting B cells. In this context, they may represent a progenitor population that eventually gives rise to malignant MM plasma cells, as proposed elsewhere (Pilarski, Reiman, Pilarski, Orr, and Belch, 2004).

Given the potential progenitor status of CD20+ MM B cells, therapeutic ablation of both compartments could be clinically valuable. In combination with conventional therapy to target the plasma cell compartment in MM bone marrow, anti-CD20 might therefore prolong survival. Targeting therapy to the CD20 epitope would be expected to deplete both the monocytoid B cells which have a moderate density of CD20 and the Leu12+ B cells which have a high density of CD20. Although Rituximab may deplete CD20+ plasma cells resident in the BM (Treon, Pilarski, Belch, Shima, Szczepek, Raje, Hideshima, Chauhan, Tau, Davies, Preffer, and Anderson, 2002), unexpectedly, treatment with Rituximab did not deplete circulating CD20^+^ B cell compartments in MM patients. Our study included phenotyping of every patient before and after infusion of rituximab for each weekly cycle as well as at monthly samples taken at 2–5 months post-treatment (over 10 sequential blood samples per patient). For all patients, transient reductions occurred in the % of circulating B cells, but for most patients, the number of MM B cells transiently or persistently increased, with a substantial % of total B cells at the end of the study period. Although the flow cytometric methods used were not quantitative, staining with anti-CD20 includes cells with both moderate and high intensity of antibody binding. If treatment with rituximab had resulted in a reduced density of CD20 epitopes, these cells would have been only weakly detectable with the methods used. However, this is unlikely to be a significant factor in the present study because CD20+ B cell % and the intensity of staining with anti-CD20 after treatment were comparable to pretreatment values. Based on our phenotypic data, rituximab does not reliably deplete circulating B cells in MM patients, and by extrapolation would not be expected to have an impact on putative MM B cell progenitor function. In addition, for 5/6 patients, CD20^+^ circulating plasma cells persisted in undiminished numbers for at least 4 months after rituximab treatment, indicating their prolonged resistance to four cycles of anti-CD20 mediated cell killing. The extent to which differing pharmacokinetics may influence the impact of rituximab therapy and the potential efficacy of larger therapeutic doses remain unknown. The mechanism(s) underlying the failure of rituximab to clear these cells may also reflect physiological properties of the normal and malignant B cells in MM patients and possibly resistance to immune effector mechanisms shown by others to mediate rituximab killing in vitro (Golay et al. 2001; Lefebvre et al. 2006; Treon et al. 2001b; van Meerten et al. 2006). Since the density of CD20 appears to be an important factor in rituximab mediated cell killing (Golay, Lazzari, Facchinetti, Bernasconi, Borleri, Barbui, Rambaldi, and Introna, 2001; van Meerten, van Rijn, Hol, Hagenbeek, and Ebeling, 2006), at least the CD20^bright^ B cells should have been depleted. Despite this, we found that CD20^bright^ B cells persisted in rituximab-treated MM patients. Rituximab has been shown to promote apoptosis in malignant cells (Bonavida, 2007; Byrd et al. 2002), but the survival of B cells in MM suggests this mechanism was not operative. Since rituximab binds to both subsets of MM B cells, the failure to deplete them may indicate that components of myeloma serum protect them from the lytic or apoptotic effects of the antibody. The functional capabilities of the MM B cells that resist rituximab have not been explored, but their persistence over several months suggests that they remain viable. It is possible that combination therapies may enhance the sensitivity to rituximab of persisting B cells. Overall, our observations suggest that the impact of rituximab in MM is different from that in lymphoma (Maloney, 2003), B-CLL (Cvetkovic and Perry, 2006; Kharfan-Dabaja et al. 2007) or Waldenstrom’s macroglobulinemia (Treon et al. 2001a). In MM, nearly all compartments of CD20+ B lineage cells have been shown here to resist the toxic effects of this serotherapy, providing a plausible explanation for the lack of clinical effect for single agent rituximab in MM (Treon, Pilarski, Belch, Shima, Szczepek, Raje, Hideshima, Chauhan, Tau, Davies, Preffer, and Anderson, 2002).

## Conclusions

The MM clone includes multiple compartments of clonotypic B cells at multiple stages of the phenotypically defined B lineage differentiation pathway. We have identified two distinct compartments of CD20+ MM B cells, both of which resist chemotherapy and are shown here to also resist the effects of rituximab. Novel therapeutic strategies are needed to target these compartments. Although the mechanisms by which rituximab successfully depletes lymphoma and other CD20+ malignancies do not appear to affect CD20+ B cells or circulating CD20+ plasma cells in MM, use of other agents to target CD20 on the MM B cell compartments, for example radiolabeled anti-CD20 (Zevalin) or a radiolabeled anti-CD19, might prove useful in MM. Because the compartments of the MM clone are quite heterogeneous and lack shared vulnerabilities, it seems likely that strategically designed combinations of two or more therapies will be needed to prolong survival by more effectively depleting the entirety of the MM clone, inclusive of multiple progenitor compartments and end stage MM plasma cells.

## Figures and Tables

**Table 1:** CD19 epitope distribution on circulating B cells in MM PBMC.

	% of PBMC expressing CD19 Epitopes		
**Source of PBMC (n)**	**B4**	**FMC63**	**Leu12**
Healthy donors (5)	7 ± 4	7 ± 3	6 ± 4
MM patients (12)	30 ± 6	27 ± 5	2 ± 1
**Patient PBMC tested at different stages of treatment (n)**†			
Diagnosis (6)			3.4 ± 3
Intermittent chemotherapy* (52)			4.1 ± 0.6
Off treatment (29)			6.7 ± 1.7
Terminal disease (10)			2.1 ± 1

Values represent the mean ± SE.

n = number of donors tested.

* For those patients on intermittent chemotherapy, blood samples were taken at least 3 weeks after their last cycle.

† Values for staining with B4 or FMC63 were comparable to those shown in the top section of this table and have been published previously (Bergsagel, Masellis, Szczepek, Mant, Belch, and Pilarski 1995); they did not differ among patients at differing stages of treatment.

**Table 2:** CD19+ B cells and CD138+ plasma cells in MM PBMC resist treatment with rituximab (anti-CD20).

Timepoint (n)	% of PBMC		
	Total CD19+ B cells	Total CD138+ PC	CD138+20+ PC
Pre-treatment (6)	19.9 ± 4.5	0.4 ± 0.15	0.26 ± 0.14
1–2 months (10) after initial treatment	17.3 ± 2.6		
3–5 months (9) after initial treatment	29.3 ± 6.8		
3–5 months (8) after initial treatment		0.38 ± 0.09	0.23 ± 0.15

Values represent the mean % ± SE for the indicated subset of PBMC.

n is the number of blood samples analyzed from the 6 patients receiving Rituximab treatment. The values include 1–2 samples per patient, averaged for each patient having more than one sample before inclusion in the table, taken within the time window indicated. Absolute numbers are provided in Table 4. PC = plasma cells.

PBMC taken from patients that had been treated with rituximab at the indicated time before or after treatment were stained with CD19 (FMC63), CD20 and CD138 or isotype matched control antibodies. The percent of CD19+ B cells indicted in the table is the aggregate number, including both includes both monocytoid and lymphocyte scatter gates. Staining with anti-CD19 was used to ensure detection of all B cells including any that had bound rituximab thereby obscuring their CD20 epitopes. Overall, the % of CD19+ B cells was comparable to the % of CD20+ B cells (as shown in Table 3) indicating that the extent of surface rituximab binding was likely to be minor. Plasma cells were identified as CD138+ cells with or without concomitant CD20 staining.

**Table 3:** Population dynamics of lymphocyte and monocytoid CD20+ B cell subsets during treatment with rituximab: Percent in PBMC.

A. Lymphocytic B cells (CD20^hi^)						
Patient	Percent of small CD20+ B cells in MM PBMC					
	#1	#2	#3	#4	#5	#6
Pre-therapy	7.9	3.2	0.4	0.5	0.9*	5.4
Week 1	0.8	10.2	7.8		2.3	0.1*
Week 2	0.9*	0.5*		1.2	0.1	0.1*
Week 3	0.1*	1.0*	10.1	0.4		
Week 4	0.01*		0.02		0.9	0.1*
1 Month		5.1*	8.4	1.4		
2 Months	4.1*		4.7	1.3	0.6	0.7
3 Months	8.9*			1.1	0.5	
4 Months	11.5	4.6*	10.2	0.2	0.4	0.6
5 Months			57.3†			

B. Monocytoid B cells (CD20^med^)						
Patient	Percent of large CD20+ B cells in MM PBMC					
	#1	#2	#3	#4	#5	#6
Pre-therapy	21.9	8.1	31.7	3.6	12.8	7.7*
Week 1	8.6*	22.7	20.0		10.0	14
Week 2	30.1*	4.6		3.6	10.9	14
Week 3	30.4	9.2	29.7	2.8		
Week 4	16.8		0.7*		19.6	18*
1 Month		10.7	15.3	1.2		
2 Months	23.0		5.3	1.4	16.5	22
3 Months	15.7			3.1		15.7
4 Months	23.5	14.6	20.3	20.3	5.5	15*
5 Months			3.4			

PBMC taken at the indicated timepoints before, during and after therapy were stained in separate reactions with anti-CD20 or with anti-CD19 (FMC63). Files were analyzed for the number of CD20+ cells in the small lymphocyte subset or in the large monocytoid B cell subset.

* Indicates samples in which the number of CD19+ B cells exceeded the number of CD20+ B cells in one or both subsets, likely indicating that CD20 epitopes had been hidden by circulating rituximab in vivo. Those samples not indicated by the asterix had comparable numbers of B cells as defined by either CD20 or CD19.

† The substantial number of small B cells seen in this sample was also detected by anti-CD19.

**Table 4:** Population dynamics of CD20+ B cells during treatment with rituximab: Absolute number in circulation.

Patient	CD20+ B cells x 10^9^/liter of blood					
	#1	#2	#3	#4	#5	#6
Pre-therapy	0.47	0.16	0.46	0.10	0.60	0.38
Week 1	0.15	0.42	0.25		0.48	0.37
Week 2	0.47	0.07		0.14	0.43	0.30
Week 3	0.43	0.12	0.44	0.10		
Week 4	0.24		0.08		0.20	0.35
1 Month		0.10	0.35	0.05		
2 Months	0.30		0.31	0.03	0.75	0.53
3 Months	0.33			0.05	0.54	
4 Months	0.56	0.15	0.24		0.13	0.43
5 Months			0.36			

The absolute number of B cells was calculated as the number of lymphocytes plus the number of monocytes (from the differential count performed by the clinical testing laboratory) multiplied by the % of PBMC that were shown to be CD20+ by flow cytometry, as presented in Table 3. The number of lymphocytes and monocytes was added to mimic the population of cells found in PBMC.
